# INFLUENCES OF CULTURAL VALUES ON THE INDIGENOUS DEMENTIA CAREGIVERS’ EXPERIENCE

**DOI:** 10.1093/geroni/igad104.0810

**Published:** 2023-12-21

**Authors:** Steffi Kim, Jordan Lewis

## Abstract

This symposium will outline and discuss the unique circumstances and challenges of American Indian, Alaska Native, and Native Hawaiians and Pacific Islander dementia caregivers. Dementia is one of the top causes of death worldwide, with Alzheimer’s disease currently being the ninth leading cause of death among American Indian and Alaska Native people (AI/AN). One in three American Indians and Alaska Natives over 65 will receive a dementia diagnosis in the next 25 years. Yet, we know very little about caregivers’ experiences in these communities. Presenters will present on how cultural influences shape caregiver’s experiences within American and Alaska Native, Native Hawaiians and Pacific Islanders. The second presenter will discuss the need for culturally safe dementia caregiver resources based on recommendations from Alaska Native caregivers. The third presenter will examine decision-making processes of dementia caregiver involved in the use of governmental and tribal dementia care resources within the ONEIDA Nation to improve utilization of existing services.

